# Interplay between CCR7 and Notch1 axes promotes stemness in MMTV-PyMT mammary cancer cells

**DOI:** 10.1186/s12943-017-0592-0

**Published:** 2017-01-31

**Authors:** Sarah T. Boyle, Krystyna A. Gieniec, Carly E. Gregor, Jessica W. Faulkner, Shaun R. McColl, Marina Kochetkova

**Affiliations:** 1 Centre for Cancer Biology, University of South Australia and SA Pathology, Adelaide, SA Australia; 2 Department of Molecular and Cellular Biology, School of Biological Sciences, University of Adelaide, Adelaide, SA Australia

**Keywords:** Breast cancer, Mammary gland, Chemokine receptor, CCR7, Notch, Cancer stem cell, Crosstalk

## Abstract

**Background:**

Breast cancer is the major cause of cancer-related mortality in women. It is thought that quiescent stem-like cells within solid tumors are responsible for cancer maintenance, progression and eventual metastasis. We recently reported that the chemokine receptor CCR7, a multi-functional regulator of breast cancer, maintains the stem-like cell population.

**Methods:**

This study used a combination of molecular and cellular assays on primary mammary tumor cells from the MMTV-PyMT transgenic mouse with or without CCR7 to examine the signaling crosstalk between CCR7 and Notch pathways.

**Results:**

We show for the first time that CCR7 functionally intersects with the Notch signaling pathway to regulate mammary cancer stem-like cells. In this cell subpopulation, CCR7 stimulation activated the Notch signaling pathway, and deletion of CCR7 significantly reduced the levels of activated cleaved Notch1. Moreover, blocking Notch activity prevented specific ligand-induced signaling of CCR7 and augmentation of mammary cancer stem-like cell function.

**Conclusion:**

Crosstalk between CCR7 and Notch1 promotes stemness in mammary cancer cells and may ultimately potentiate mammary tumor progression. Therefore, dual targeting of both the CCR7 receptor and Notch1 signaling axes may be a potential therapeutic avenue to specifically inhibit the functions of breast cancer stem cells.

**Electronic supplementary material:**

The online version of this article (doi:10.1186/s12943-017-0592-0) contains supplementary material, which is available to authorized users.

## Introduction

Despite significant recent advances in breast cancer treatment, breast cancer is still the major cause of cancer-related death in females worldwide. Resistance to radiation and chemotherapy, together with unpredictable recrudescence, are the most challenging issues in clinical practice. Significant evidence has accumulated in recent years to implicate cancer stem-like cells (CSCs) as the main perpetuators of cancer recurrence and therapy resistance in mammary and other tumors, as this cell population exhibits stem cell-like characteristics of self-renewal, quiescence and the ability to differentiate into various cell lineages [1, 2]. Therefore, targeting alterations acquired by CSCs in stemness-related signaling pathways has been proposed as an effective therapeutic strategy to counteract current treatment shortfalls in breast cancer management. However, CSC regulatory mechanisms remain largely undiscovered.

Chemokine receptors and their ligands have been implicated in numerous aspects of breast malignancy [3]. The chemokine receptor CCR7 and its ligands CCL19 and CCL21 have been associated with metastasis, poor survival, invasion and cell migration in primary tumors and breast cancer experimental models [4–6]. We have recently shown that CCR7 regulates stem cell-like activity in primary mammary tumors in mice and humans [7], and sought to understand how CCR7-mediated stemness interacts with and/or is influenced by recognized stem cell-related signaling pathways.

The Notch pathway is one of the major signaling axes in embryonic and adult stem cell homeostasis and has a critical role in regulating proliferation, differentiation, apoptosis, cell-cell communication and other multiple functions [8, 9]. Notch receptors are proteolytically cleaved in a two-step process, releasing the intracellular domain that translocates to the nucleus and acts as a transcriptional cofactor for various genes including those mediating cell fate [10]. A cancer-specific role of the Notch axis has also been extensively studied in both solid and blood neoplasms, and numerous inhibitors of γ-secretase – the enzyme that cleaves and hence activates Notch signaling — are now being trialed in human patients as anti-cancer drugs [11, 12]. Notably, Notch has been reported to play both oncogenic and tumor-suppressive roles depending on the cell type and tissue context [10], thus warranting further investigation into molecular determinants of the tumor-promoting or tumor-inhibiting arms of this complex, multifaceted signaling pathway.

Signaling through the Notch axis has been reported to crosstalk with multiple other pathways including numerous oncogenic mediators, such as Wnt effectors [13], the Hedgehog pathway, cytokines, kinases and growth factors in mammary and other cancers [14, 15]. Of note is the intersection between the epidermal growth factor receptor (EGFR) system and Notch pathway that has been proposed as a mechanism for trastuzumab resistance in HER2-positive tumors, and thus a route for therapeutic intervention [16]. Effectively, the identification of specific crosstalk networks of Notch that govern growth and differentiation of mammary cancer cells may provide new opportunities for developing effective inhibitors of tumor relapse and metastasis.

As Notch pathway members have been implicated in maintenance of stem cell pools within mammary tumors [17–19], and having previously found that activation of CCR7-mediated signaling also promotes stemness [7], we hypothesized that these pathways may interact within breast CSC pools to enhance and maintain tumor cell stemness properties. Using the MMTV-PyMT mouse model, we show here that CCR7 and Notch1 cooperate and crosstalk within transformed mammary stem cell-like compartments, indicating that the CCR7-Notch axis may be a potential target for therapeutic intervention in breast cancer.

## Materials and methods

### Mice

MMTV-PyMT; *Ccr7* ^WT^ (referred to as PyMT-*Ccr7* ^WT^) and MMTV-PyMT; *Ccr7* ^−/−^ (referred to as PyMT-*Ccr7* ^−/−^) mice on a pure C57BL/6 background were generated and described previously [7]. The University of Adelaide institutional animal ethics committee approved all protocols.

### Mammary cell extraction

Mammary epithelial cell extraction was performed as described [20]. Briefly, mouse mammary glands/tumors were dissected, manually dissociated and then digested in Dulbecco’s modified Eagle’s medium (DMEM, Gibco) supplemented with 1 mg/ml collagenase, 100U/ml hyaluronidase (both from Worthington) and 2% foetal calf serum for 3 h at 37 °C. Resultant organoids were digested a further 15 min with 6U/ml dispase (Gibco) and 12U/ml DNase I (Merck) at 37 °C. Cells were filtered through a 70 μm nylon mesh to obtain a single cell suspension.

### Mammosphere culture

Mammosphere cultures were established by seeding freshly extracted mammary cells in complete sphere medium (1:1 volumes of DMEM and Ham’s F12 nutrient mix (Gibco) supplemented with 1× B27 (Invitrogen), 10 ng/ml epidermal growth factor (R & D Systems), 20 ng/ml basic fibroblast growth factor (R & D Systems), 4 ng/ml heparin (Sigma Aldrich), and 1% penicillin-streptomycin) to polyhema-coated plates. After 7 days, mammospheres were passaged by centrifuging at 200xg for 5 min, resuspended in trypsin/EDTA and triturated through a 19G needle to break up colonies. Cells were filtered through 70 μm and washed extensively before re-seeding for secondary culture in complete sphere medium. For all assays, secondary cultures were analyzed. Where specified, spheres were cultured in the presence of γ-secretase inhibitor RO4929097 (SelleckChem) at 50nM, Wnt/β-catenin inhibitor XAV-939 (SelleckChem) at 1 μM, CCL19 at 200 ng/ml, and CCL21 at 10 ng/ml. For analysis of mammosphere-forming efficiency, secondary spheres were assessed for number of spheres formed per cells seeded.

### Flow cytometry and FACS

Flow cytometry/FACS was carried out by blocking for non-specific binding for 15 min at room temperature in mouse γ-globulin in PBS/0.5% bovine serum albumin (BSA) followed by immunostaining for 30–45 min on ice in PBS/0.5% BSA. Primary anti-mouse antibodies used were anti-Notch1ECD-APC, biotinylated anti-Notch1ICD (eBioscience), anti-CD24-PE, anti-CD29-PE, anti-CCR7-AlexaFluor647 (BD Biosciences), anti-CD24-FITC, anti-CD24-PE/Cy5 (Life Technologies), anti-CD29-FITC, anti-CD29-AlexaFluor647, anti-Jagged1-PE and anti-DLL1-AlexaFluor647 (BioLegend). For Notch1ICD, cells were permeabilized prior to labelling followed by detection using streptavidin-AlexaFluor488 (BD Biosciences) in PBS/0.5% BSA for 30 min on ice. Fluorescence-minus-one (FMO) controls for each antigen in multi-color analysis were used for negative gating. MFI = mean fluorescence intensity.

### Western analysis

Proteins were separated by 10% SDS-PAGE and immobilized onto nitrocellulose membrane. Western analysis was performed as previously described [21]. The γ-secretase inhibitor RO4929097 (SelleckChem) was used at 50nM and CCL19 at 200 ng/ml and times of treatment are specified in the figures and figure legends. Primary antibodies used were anti-Notch1 ICD (Abcam), anti-Hes1 (R & D Systems), anti-phospho-ERK, anti-ERK1/2 (both from Cell Signaling Technology) and anti-β-Actin (Sigma-Aldrich). Band intensities were calculated using Image J.

### Cyclic AMP analysis

cAMP levels were measured using an AlphaScreen cAMP Assay Kit (Perkin-Elmer) as per manufacturer’s instructions. The γ-secretase inhibitor RO4929097 (SelleckChem) was used at 50nM, CCL19 at 200 ng/ml, and CCL21 at 10 ng/ml. Cells were treated for 30 min before analysis.

### mRNA analysis

Total RNA was extracted using an RNeasy Kit (Qiagen) and reverse-transcribed using the Transcriptor First Strand Kit (Roche), followed by quantitative PCR analysis using the SYBR Green method. Samples were normalized to endogenous ribosomal protein *Rpl32*. qPCR primer sequences are as follows:

**Table:** 

Gene	Forward Primer 5′–3′	Reverse Primer 5′–3′
*Hes1*	TCCAAGCTAGAGAAGGCAGAC	TGATCTGGGTCATGCAGTTG
*Ccr7*	CATTGCCTATGACGTCACCTACA	GAAGGCATACCAGAAAGGGTTGA
*Ccl19*	CTGCCTCAGATTATCTGCCAT	CTTCCGCATCATTAGCACCC
*Ccl21*	GCAAAGAGGGAGCTAGAAAACAGA	TGGACGGAGGCCAGCAT
*Rpl32*	AAGCGAAACTGGCGGAAAC	TAACCGATGTTGGGCATCAG

### Statistics

Data are mean ± SEM and results are representative of at least 3 independent experiments. Significant statistical differences were estimated using Student’s t-tests or ANOVA and significant associations between CCR7 and Notch1 were identified using Pearson correlation coefficients. *P*-values were used to denote statistical significance: ^*^ *p* ≤ 0.05, ^**^ *p* ≤ 0.01, ^***^ *p* ≤ 0.001 and ^****^ *p* ≤ 0.0001.

## Results

### CCR7 activates the Notch1 signaling pathway in mammary cancer stem-like cells

**Fig. 1:**
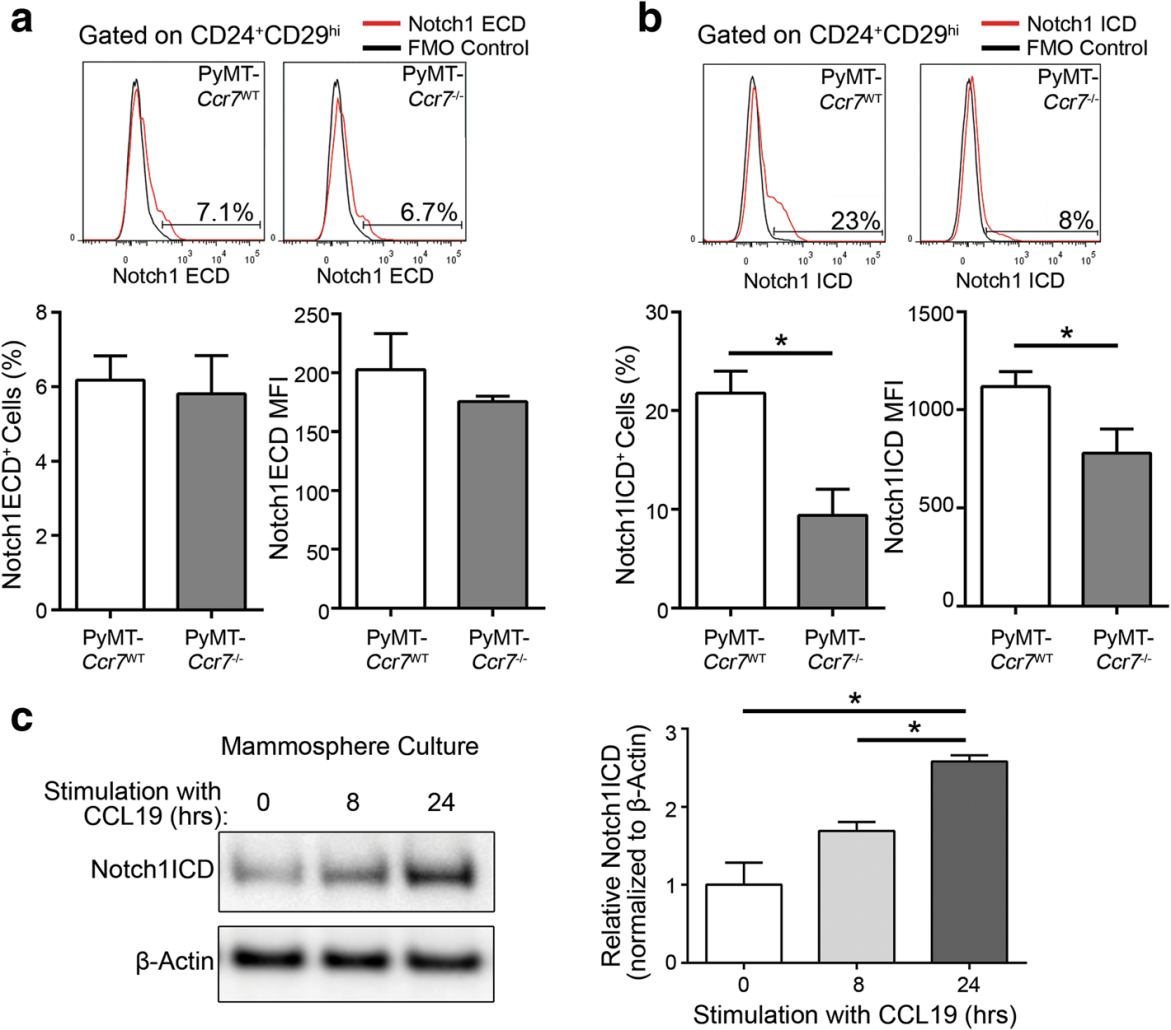
Cleavage of Notch1 in mammary cancer stem-like cells is dependent on CCR7. **a**-**b** Primary PyMT-*Ccr7* ^WT^ and *Ccr7* ^−/−^ mammary tumor cells were analyzed by multi-color flow cytometry for Notch1 expression within the stem cell-enriched population CD24^+^CD29^hi^. Top = representative flow cytometry plots, bottom = quantification. **a** Notch1 extracellular domain (ECD) cell surface expression in the PyMT-*Ccr7* ^WT^ and *Ccr7* ^−/−^ mammary stem cell-like population. **b** Notch1 intracellular domain (ICD) levels in the PyMT-*Ccr7* ^WT^ and *Ccr7* ^−/−^ mammary stem cell-like population. Bulk tumor cells were first labelled for extracellular CD24 and CD29 before permeabilization and staining for Notch1 ICD. **a**-**b** *n* = 4 mice/genotype. FMO = fluorescence minus one, MFI = mean fluorescence intensity. **c** Notch1 ICD levels in PyMT-*Ccr7* ^WT^ secondary mammospheres, stimulated with CCL19 for varying lengths of time as indicated before lysis for Western analysis. *n* = 6 mice/experiment

In the search for mechanistic clues of the CCR7-mediated augmentation of the mammary stem cell compartment, we investigated crosstalk between CCR7 and Notch1 pathways, as Notch1 signaling has also been implicated in mammary cancer stem cells [17]. The Notch intracellular domain is released upon receptor activation and acts as a transcriptional cofactor to promote stemness [10], therefore we examined the levels of Notch1 receptor domains in the CD24^+^CD29^hi^ stem cell-enriched population [22, 23] from PyMT-*Ccr7* ^WT^ and PyMT-*Ccr7* ^−/−^ murine mammary tumors. The CD24^+^CD29^hi^ gating strategy we have employed for all multi-color flow cytometry throughout the study is shown in Additional file 1: Figure S1a. While CCR7 did not affect cell-surface expression of Notch1 (Fig. 1a), levels of the Notch1 intracellular domain (N1ICD) were significantly reduced in CCR7-null stem-like cells (Fig. 1b). This finding was substantiated by results obtained in secondary mammospheres (that are enriched for stem and progenitor cells [24]) from PyMT-*Ccr7* ^WT^ tumors, as stimulation of CCR7 with its ligand CCL19 significantly increased levels of N1ICD over time (Fig. 1c). Expression of common Notch ligands DLL1 and Jagged1 was not affected by CCR7 (Additional file 1: Figure S1b and c), and hence findings were not due to an alteration of Notch ligands on CSCs, but rather Notch1 itself. Taken together, these data indicate that Notch1 cleavage is significantly potentiated by CCR7.

**Fig. 2:**
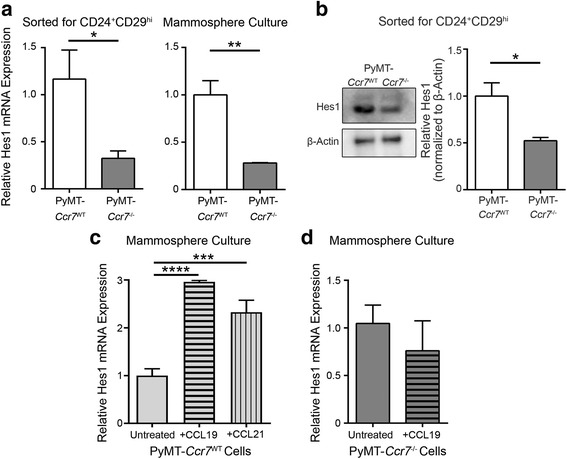
CCR7 activates the Notch signaling pathway in mammary cancer stem-like cells. **a**-**b** Hes1 expression was assessed in primary mammary CSCs from PyMT-*Ccr7* ^WT^ and *Ccr7* ^−/−^ tumors. **a** Hes1 mRNA levels relative to PyMT-*Ccr7* ^−/−^ in the sorted CD24^+^CD29^hi^ stem cell-enriched population (*left*) and in secondary mammospheres (*right*), *n* = 4–6 mice/genotype/experiment. **b** Hes1 protein expression in the CD24^+^CD29^hi^ sorted cell population, *n* = 4 mice/genotype. **c** Relative Hes1 mRNA levels in PyMT-*Ccr7* ^WT^ secondary mammospheres cultured with and without CCL19 and CCL21. **d** Relative Hes1 mRNA levels in PyMT-*Ccr7* ^−/−^ secondary mammospheres cultured with and without CCL19. **c**–**d** *n* = 6 mice/experiment

To establish if CCR7-mediated regulation of the Notch pathway is functional, we tested the levels of Notch transcriptional target Hes1 [10]. Both Hes1 mRNA and protein levels were significantly reduced in CCR7-null cancer stem cell-like populations (Fig. 2a and b), either sorted for CD24^+^CD29^hi^ expression by FACS or selected through culturing as secondary mammospheres. Importantly, direct stimulation of PyMT-*Ccr7* ^WT^ mammospheres through culturing in the presence of either CCL19 or CCL21 greatly increased Hes1 expression (Fig. 2c), validating results obtained with the CCR7-null cells. Furthermore, Hes1 regulation by CCR7-cognate chemokines was receptor specific as it was not seen in PyMT-*Ccr7* ^−/−^ cells (Fig. 2d). These results suggest that CCR7 regulates expression of Notch target stemness-promoting genes, and thus CCR7-mediated functional properties of stemness may involve Notch pathway activity.

### Notch mediates CCR7-promoted stemness in mammary cancer cells

As Notch activation requires proteolytic cleavage by γ-secretase, we utilized the γ-secretase inhibitor RO4929097 (previously used in breast cancer studies to inhibit Notch signaling [25]) to examine the effect of CCR7 on mammary cancer stem cells in the context of Notch activity blockade. The effect of RO4929097 in secondary PyMT-*Ccr7* ^WT^ mammospheres was first verified by examining Hes1 expression, which was significantly reduced by addition of the inhibitor (Additional File 1: Figure S2a). Notably, we found that treatment with RO4929097 did not affect expression of CCR7 or its ligands CCL19 and CCL21 (Additional File 1: Figure S2b–d), in contrast with other studies in leukemic cells [26].

**Fig. 3:**
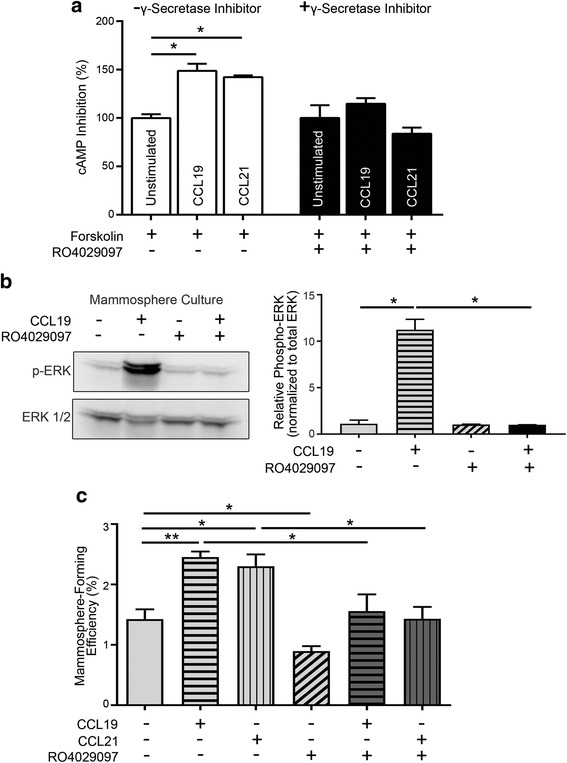
Notch promotes CCR7-mediated stemness. **a** Level of cyclic AMP (cAMP) in PyMT-*Ccr7* ^WT^ mammospheres with and without CCL19 stimulation and Notch inhibition, determined by an AlphaScreen cAMP assay. Cells were treated with CCL19, CCL21 and RO4029097 for 30 min before analysis. **b** PyMT-*Ccr7* ^WT^ secondary mammospheres were tested for phosphorylation of ERK as a read-out for CCR7 activation with and without ligand stimulation and Notch inhibition. Cells were treated with CCL19 and RO4029097 for 15 min before lysis for Western analysis. **c** Secondary mammosphere-forming efficiency (number of spheres/cells seeded) of PyMT-*Ccr7* ^WT^ primary mammary tumor cells cultured with and without chemokines CCL19 and CCL21, and RO4029097. **a**–**c** *n* = 6 mice/experiment

We then assessed the effect of RO4929097 on the function of CCR7 in mammary stem-like cells. We found that inhibition of cyclic AMP (cAMP), a read-out for chemokine receptor activity but not a known outcome of Notch signaling, was induced by CCL19 and CCL21; however addition of RO4929097 abrogated this chemokine-mediated inhibition of forskolin-induced cAMP (Fig. 3a).

To further confirm that the CCR7 pathway interacts with Notch signaling, we analyzed mammosphere growth after addition of CCL19 and CCL21 with and without RO4929097. We observed that blocking γ-secretase significantly inhibited CCL19-mediated phosphorylation of ERK, an effector of CCR7 signaling that is not downstream of Notch itself (Fig. 3b). Notch also affected the ability of CCR7 to stimulate mammosphere formation. In concordance with a previous study [27] RO4929097 reduced secondary mammosphere formation. The addition of CCL19 or CCL21 was not sufficient to restore mammosphere formation to levels seen with chemokine stimulation alone (Fig. 3c), suggesting that inhibition of Notch inhibits the stemness-promoting function of CCR7. However, mammosphere formation was partially rescued, indicating that there are other Notch-independent pathways in play in this context.

We observed that the other major signaling pathway previously implicated in stem cell maintenance, Wnt/β-catenin [28], did not impact upon CCR7-mediated stemness as the Notch pathway appears to. Specifically, while inhibition of Wnt signaling with the specific inhibitor XAV-939 significantly decreased sphere-forming ability and hence stemness properties in primary PyMT-expressing tumor cells as expected [29], addition of the CCR7 ligand CCL21 to XAV-939 treated cells was sufficient to restore sphere formation to that seen when stimulated with CCL21 alone (Additional File 1: Figure S3), thus demonstrating that the Wnt pathway is not involved in CCR7-mediated regulation of stem cell maintenance.

These data show that the function of CCR7 in promoting the stemness of mammary tumor cells is partly mediated through Notch signaling. Although CCR7 regulates Notch1 activation in MMTV-PyMT tumor cells, the two pathways likely cooperate to promote CSC activity.

## Discussion

Despite advances in clinical treatment of breast cancer, recurrence remains a significant problem due to the survival of quiescent cancer stem cells. Novel combination therapies against CSCs, acting as adjuvants together with conventional approaches, are therefore needed. We previously demonstrated that the chemokine receptor CCR7 promotes mammary tumorigenesis via amplification of stem-like cells [7], and we now show here that crosstalk between Notch and CCR7 signaling promotes breast cancer stemness and may be a potential target for these new therapies.

Notch can have opposing roles depending on biological context, with Notch1 activation shown to both perpetuate and inhibit cell differentiation [10]. Notch also regulates, and is regulated by, different chemokine receptors. In acute lymphoblastic leukemia, Notch1 regulates cell proliferation and migration through CCR5 and CCR9 [30], and in multiple myeloma Notch regulates CXCR4 [31]. While no functional crosstalk between the two pathways has been reported so far, it was suggested that Notch activation up-regulates CCR7 expression in leukemic cells [26]. However, in TCR-activated peripheral blood mononuclear cells CCR7 expression was slightly increased following Notch inhibition with RO4929097 [25]. In breast cancer, CCL2 secreted from cancer-associated fibroblasts induces a CSC phenotype by activating Notch signaling, highlighting the concept that crosstalk may exist between the two axes [32]. Plausibly, the interplay between CCR7 and Notch is adaptable in different systems.

In this study, we have demonstrated that the deletion of CCR7 significantly decreases levels of activated Notch1, and conversely, stimulation of the CCR7 pathway increases Notch receptor activation and expression of downstream target genes. We did not find any substantial Notch-mediated regulation of CCR7 receptor or ligand expression but observed a significant crosstalk at the functional level, which was not seen with the other major signaling pathway involved in stem cell maintenance, Wnt/β-catenin [28]. Notably, unlike other cellular types such as lymphocytes that display differential-ligand induced CCR7 signaling and functional events [33] the observed CCR7 effect upon stemness is not ligand-specific, as we observed similar results for both CCL19 and CCL21.

Importantly, these findings may be translatable to human disease, as analysis of a published database available in Oncomine [34] showed that expression of CCR7 and Notch1 are significantly correlated in primary human breast cancers, particularly in higher grade tumors (Additional File 1: Figure S4). Although Notch proteins are known to regulate stemness, and CCR7 to promote tumorigenesis through a number of pathways, to the best of our knowledge this is the first study linking Notch1 to CCR7-dependent regulation of stem-like cells in mammary cancer. This novel phenomenon opens up the avenue for concurrent therapeutic targeting of these pathways. Antagonists towards CCR7 are capable of reducing stem-like cell content and activity [7], and γ-secretase inhibitors and antibodies that block the Notch pathway have also been shown to specifically target tumor-initiating cells [35, 36]. This approach may therefore be a new opportunity for targeting evasive cancer stem cells to control breast cancer progression.

## Additional file

Additional file 1: Supplementary Figures. (PDF 17190 kb)
