# Supplementary material for: Interplay between CCR7 and Notch1 axes promotes stemness in MMTV-PyMT mammary cancer cells
Source: Mol Cancer. 2017 Jan 31;16:19. doi: 10.1186/s12943-017-0592-0 (PMC5282896; doi:10.1186/s12943-017-0592-0)

Additional file

Interplay between CCR7 and Notch1 axes promotes stemness in MMTV-PyMT mammary cancer cells.

Sarah T. Boyle, Krystyna A. Gieniec, Carly E. Gregor, Jessica W. Faulkner, Shaun R. McColl, Marina Kochetkova

Figure S1. CCR7 does not regulate expression of Notch ligands

(a) Representative gating strategy for the delineation of the stem cell-enriched $CD24^{+}CD29^{hi}$ population as used in this project. Within each experiment, the $CD24^{+}CD29^{hi}$ population was either analyzed further by flow cytometric analysis, or sorted by FACS from the bulk tumor to analyze by Western or qPCR. **(b-c)** PyMT-*Ccr7*^{WT} and *Ccr7*$^{-/-}$ mammary tumors were analyzed by multi-color flow cytometry for expression of Notch ligands within the $CD24^{+}CD29^{hi}$ stem cell-enriched population. Top=representative flow cytometry histograms, bottom=quantification. **(b)** Cell surface expression of Notch ligand DLL1 in the PyMT-*Ccr7*^{WT} and *Ccr7*$^{-/-}$ mammary stem cell-like population. **(c)** Cell surface expression of Notch ligand Jagged1 in the PyMT-*Ccr7*^{WT} and *Ccr7*$^{-/-}$ mammary stem cell-like population. **(b-c)** n=4 mice/genotype. FMO=fluorescence minus one, MFI=mean fluorescence intensity.

Figure S2. Notch does not regulate expression of CCR7 or its ligands in mammary cancer stem cell-enriched culture

(a) Relative mRNA levels of Hes1 in PyMT-*Ccr7*^{WT} secondary mammospheres cultured with or without γ-secretase inhibitor RO4929097 to block Notch activation. **(b)** Relative mRNA levels of CCR7 in secondary mammospheres derived from PyMT-*Ccr7*^{WT} mammary tumors, cultured with or without RO4929097. **(c)** Cell surface expression of CCR7 on secondary mammospheres derived from PyMT-*Ccr7*^{WT} mammary tumors, cultured with or without RO4929097, as assessed by flow cytometry of mammosphere cells. Top=representative flow cytometry histograms, bottom=quantification. FMO=fluorescence minus one, MFI=mean fluorescence intensity. **(d)** Relative mRNA levels of CCR7 ligands CCL19 (left) and CCL21 (right) in secondary mammospheres derived from PyMT-*Ccr7*^{WT} mammary tumors, cultured with or without RO4929097. **(a-d)** n=6 mice/experiment.

Figure S3. Wnt signaling inhibition dos not affect the ability of CCR7 to stimulate mammosphere formation.

Mammosphere-forming efficiency (number of spheres/cells seeded) of PyMT-*Ccr7*^{WT} primary mammary tumor cells cultured with and without chemokine CCL21 and Wnt inhibitor XAV-939. n=6 mice/experiment.

Figure S4. Correlation of CCR7 and Notch1 expression in primary human breast cancer.

CCR7 and Notch1 expression in various grades of breast cancer, analyzed using a published dataset available in Oncomine, consisting of 2,000 primary breast cancers. Significant associations were identified using Pearson correlation coefficients.

SUPPLEMENTARY FIGURE 1

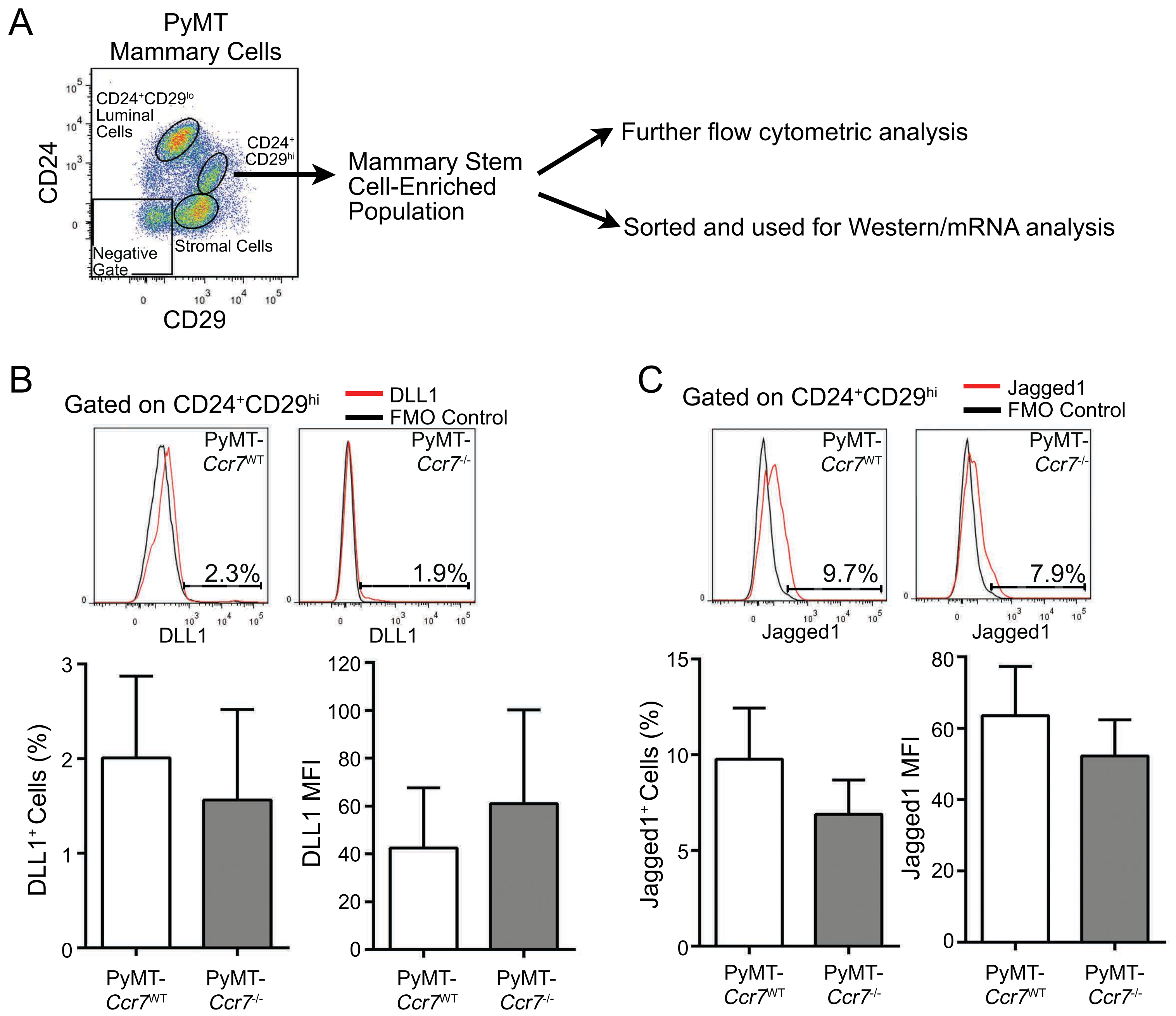

SUPPLEMENTARY FIGURE 2

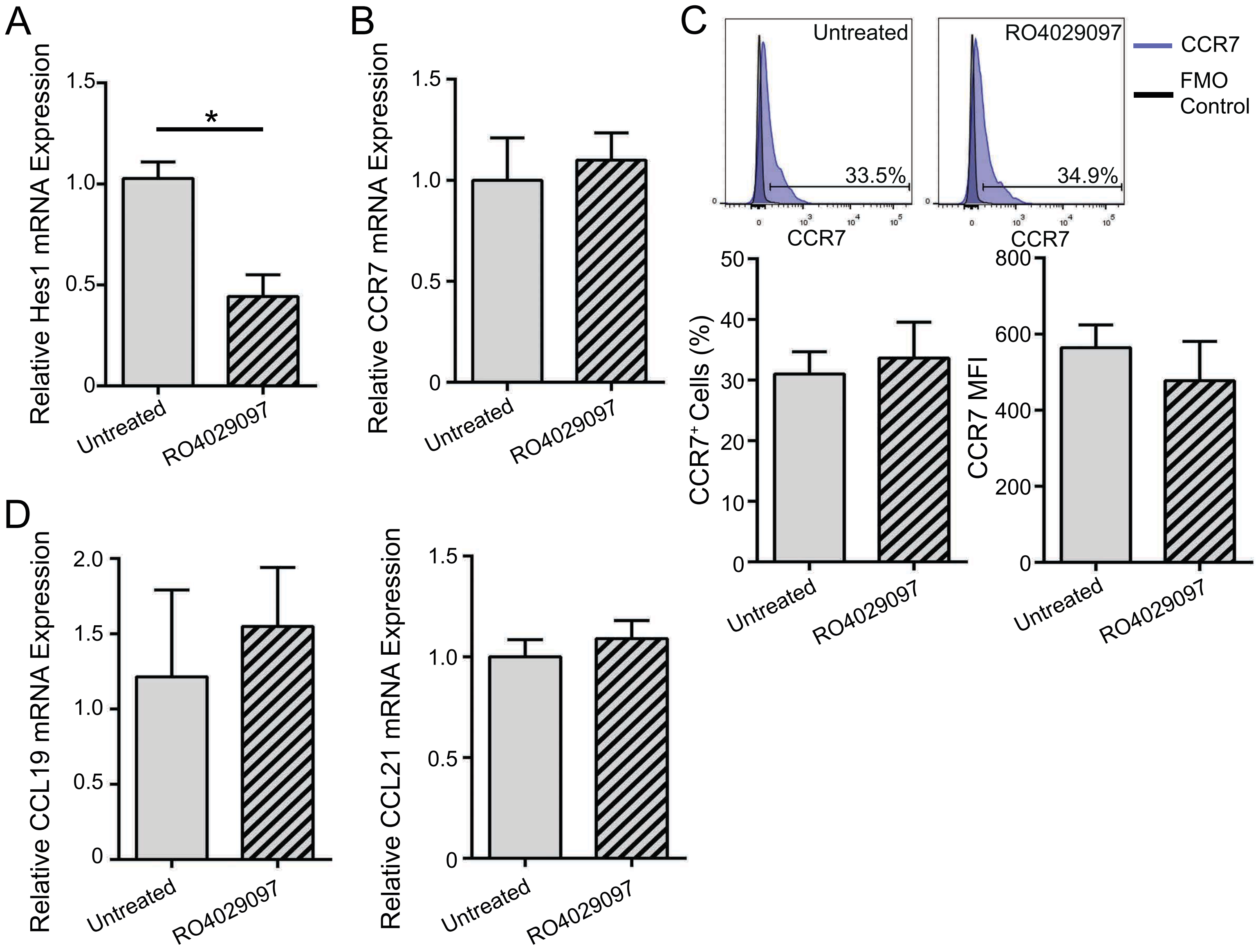

SUPPLEMENTARY FIGURE 3

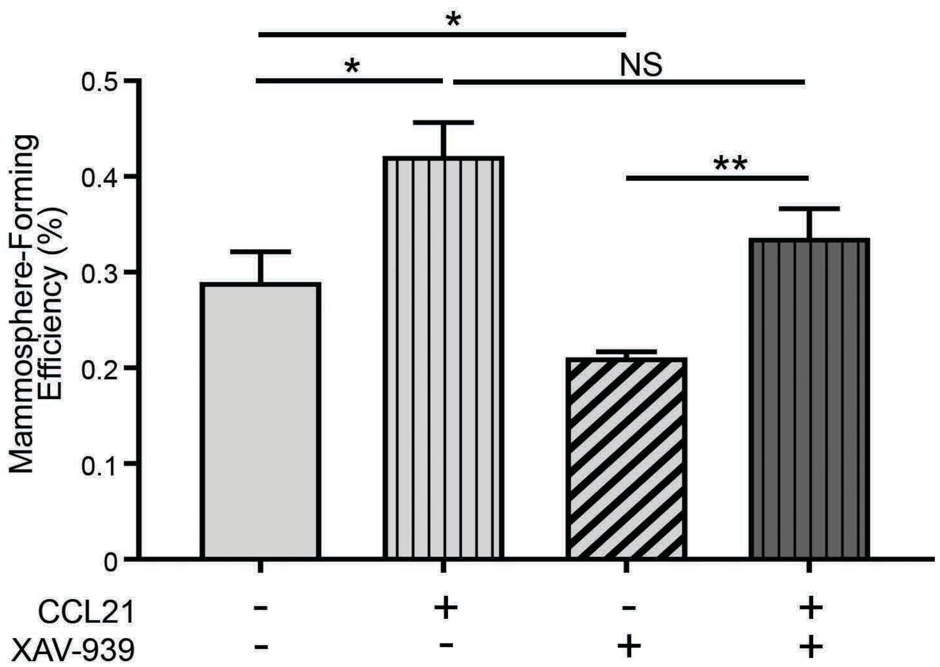

SUPPLEMENTARY FIGURE 4

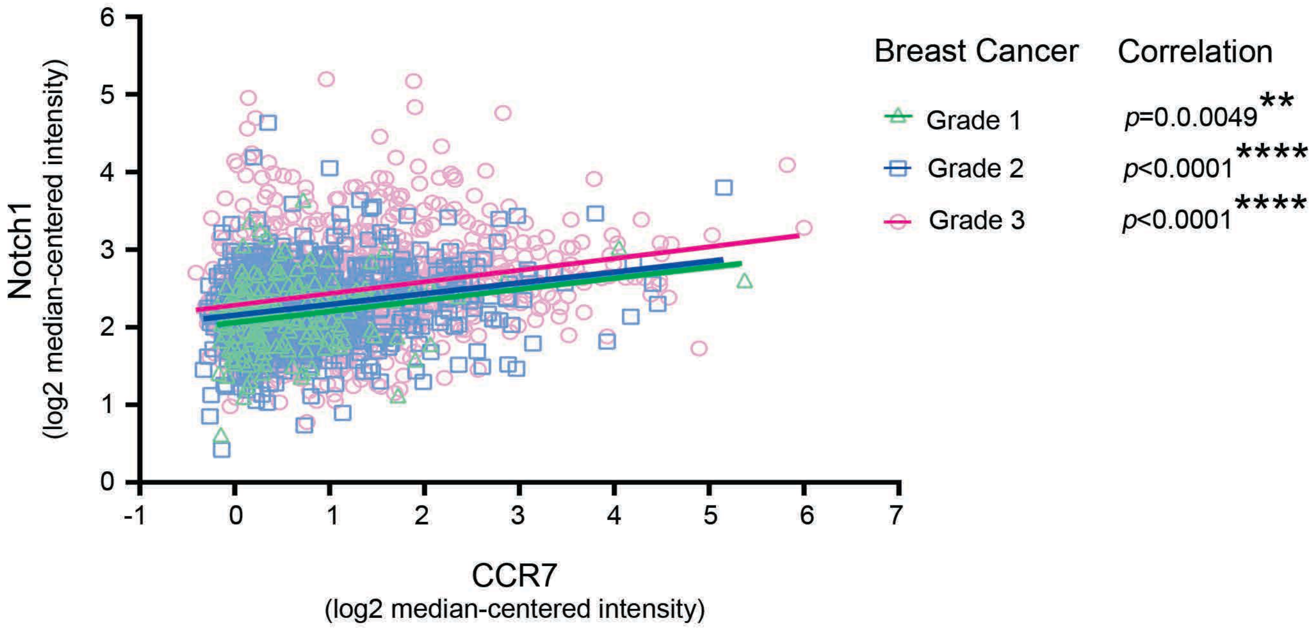

Supplement: Additional file 1: — Supplementary Figures. (PDF 17190 kb) [file 12943_2017_592_MOESM1_ESM.pdf]
